# Early qualitative analysis to enhance trial processes

**DOI:** 10.1186/1745-6215-16-S2-P73

**Published:** 2015-11-16

**Authors:** Will Storrar, Ann Dewey, Anoop Chauhan, Carole Fogg, Ellie Lanning, Thomas Brown, Lara Balls

**Affiliations:** 1 Portsmouth Hospitals NHS Trust, Portsmouth, UK; 2 University of Portsmouth, Portsmouth, UK

## 

The LASER Trial is an RCT of the effectiveness of the use of a Temperature-controlled Laminar Airflow (TLA) device in adults with severe allergic asthma. The trial is funded by the NIHR-HTA.

The trial aims to determine whether home-based nocturnal treatment with a TLA device can reduce the frequency of asthma exacerbations over a one year period. In order to inform decisions about implementation of the device should it be shown to be effective, participant's experience of using the TLA device is being assessed by means of qualitative interviews.

We conducted telephone interviews with 10 trial participants in the first 4 months of the trial in order to determine participant's thoughts, values and opinions of trial processes and the treatment device.

This early qualitative evaluation as part of the larger experimental study has provided us with an opportunity to address different questions (What is happening? How is it happening?) than otherwise would be possible with quantitative data collection alone. We have been able to provide feedback to develop and enhance subsequent trial processes including recruitment and retention of participants.

Experience from conducting this early evaluation is that the qualitative interviews and subsequent analysis have provided a useful insight into the difficulties that patients have experienced and what is important to them. As a result we have a better understanding and this has resulted in an informed plan of how to address these learning points with actions to enhance the experience of taking part in the trial for future participants.

